# Proposal for nationwide registration of injuries or fatalities in wheelchair user motor vehicle passengers in Japan

**DOI:** 10.1265/ehpm.24-00160

**Published:** 2024-08-28

**Authors:** Masahito Hitosugi, Ayumu Kuwahara, Mami Nakamura

**Affiliations:** Department of Legal Medicine, Shiga University of Medical Science, Tsukinowa, Seta, Otsu, Shiga 520-2192, Japan

**Keywords:** Wheelchair, Motor vehicle collision, Fatality, Seatbelt, Injury, Aging

## Abstract

One consequence of population aging is an increase in the number of older wheelchair users. They often board the motor vehicle from the rear for moving. Recently, wheelchair user vehicle passengers have involved in motor vehicle collisions and died. A three-point seatbelt does not adequately fit most wheelchair user passengers because of the way that the wheelchair is constructed. Therefore, owing to the movement of the body immediately after the collision, the wheelchair user passengers attacked their body to the interior of the vehicle or suffered from the intrusion of the lap belt into the abdomen, subsequently suffered from severe head, chest or abdominal injuries. According to the review of all fatal motor vehicle collisions in Shiga Prefecture, Japan, which has a population of approximately 1.4 million, from 2017 to 2022, the rate of wheelchair users in fatal motor vehicle passenger increased from 3.6% in 2017 to 2019 to 7.8% in 2020 to 2022. Therefore, there is a risk that substantial numbers of wheelchair user passengers involved in motor vehicle collisions will die. However, in Japan, there are no official statistics on the involvement of wheelchair user passengers in motor vehicle collisions. Therefore, we propose a nationwide registration of injuries and fatalities in wheelchair user passengers. Investigating the mechanisms of injury in wheelchair user passengers would contribute to the development of safety measures, especially for restraint systems. Established preventive measure would contribute to the decrease of fatally or severely injured motor vehicle collision passengers.

Dear Editors,

Road traffic collision (RTC) is one of the important issues for the health worldwide. In Japan, 2,678 persons died and 27,636 severely injured with the treatment duration of 30 days or more due to RTCs in 2023. The population aged 65 years or more accounted 54.7% of fatalities and 36.7% of severely injured persons. As these numbers has increased from a previous year, further efforts are required to keep road safety. The Japanese government enacted the Basic Law on Traffic Safety Measures in 1970 [1]. Under this law, targets have been set in the Basic Plan for Traffic Safety every 5 years since 1971, and traffic safety measures have been comprehensively and systematically promoted. The original goal of these efforts was to realize a society without RTCs. In 2021, the Japanese government established the 11^th^ Traffic Safety Basic Plan and set a new target that aims to reduce the number of RTC fatalities to less than 2,000 and the number of serious injuries to less than 22,000 by 2025 [2]. In the plan, as a basic principle, further ensuring the safety of vulnerable people such as the elderly with disabilities is specified for achieve a society in which vulnerable person can become socially independent.

In Japan, the number of older persons is increasing and persons aged 65 years or more accounted for 29.1% of the whole population in 2023. One consequence of population aging is an increase in the number of older wheelchair users. Older persons often receive some kind of welfare service, such as day care with rehabilitation, for which they must be transferred from their homes to the welfare facilities by motor vehicle. Such transportation often requires wheelchair users to board the vehicle from the rear. Recently, wheelchair user vehicle passengers have involved in motor vehicle collisions (MVCs) and subsequently died. Investigations of the causes and mechanisms underlying these deaths have shown that the fatal injuries were partly caused by inappropriate seatbelt restrictions [3]. A three-point seatbelt does not adequately fit most wheelchair user passengers because of the way that the wheelchair is constructed. Because a lap belt passes over or under the wheelchair handrails, it does not adequately fit the lumbar region of wheelchair users. Therefore, owing to the movement of the body immediately after the collision, the wheelchair user passengers attacked their body to the interior of the vehicle or suffered from the intrusion of the lap belt into the abdomen, subsequently suffered from severe head, chest or abdominal injuries. Some wheelchair users do not fasten the shoulder belt because it is positioned against the neck, which is an incorrect position. At current road traffic laws in Japan, because the wheelchair user passengers do not sit on the vehicle seat, legal requirement of seatbelt use is exempted for wheelchair user passengers. Therefore, more guidelines are needed on safety for wheelchair user vehicle passengers in Japan.

With the approval of the ethics committee of Shiga University of Medical Science (No. R2014-186), we reviewed all fatal MVCs in Shiga Prefecture, Japan, which has a population of approximately 1.4 million, from 2017 to 2022. Among 151 RTC fatalities between 2017 and 2019, 55 persons were motor vehicle passengers including two wheelchair users. Between 2020 and 2022, 51 fatal motor vehicle passengers including four wheelchair users were observed among 124 RTC fatalities. The rate of wheelchair users in fatal motor vehicle passenger increased from 3.6% in 2017 to 2019 to 7.8% in 2020 to 2022. Therefore, there is a risk that substantial numbers of wheelchair user passengers involved in MVCs will die.

However, in Japan, there are no official statistics on the involvement of wheelchair user passengers in MVCs. Therefore, we propose a nationwide registration of injuries and fatalities in wheelchair user passengers. Investigating the mechanisms of injury in wheelchair user passengers would contribute to the development of safety measures, especially for restraint systems. Japan has the largest aging population in the world; therefore, ensuring the safety of older wheelchair users during transportation is a very important issue. Furthermore, established preventive measure would contribute to the decrease of fatally or severely injured MVC passengers stated as the new target in the 11th Traffic Safety Basic Plan in Japan.

## Declarations

### Availability of data and material

The data presented in this study are available upon request from the corresponding author.
